# Insights into the Regulatory Role of Non-coding RNAs in Cancer Metabolism

**DOI:** 10.3389/fphys.2016.00342

**Published:** 2016-08-08

**Authors:** Fredy O. Beltrán-Anaya, Alberto Cedro-Tanda, Alfredo Hidalgo-Miranda, Sandra L. Romero-Cordoba

**Affiliations:** Cancer Genomics Laboratory, National Institute of Genomic MedicineMexico City, Mexico

**Keywords:** metabolic reprogramming, cancer metabolism, ncRNA regulation, miRNAs

## Abstract

Cancer represents a complex disease originated from alterations in several genes leading to disturbances in important signaling pathways in tumor biology, favoring heterogeneity that promotes adaptability and pharmacological resistance of tumor cells. Metabolic reprogramming has emerged as an important hallmark of cancer characterized by the presence of aerobic glycolysis, increased glutaminolysis and fatty acid biosynthesis, as well as an altered mitochondrial energy production. The metabolic switches that support energetic requirements of cancer cells are closely related to either activation of oncogenes or down-modulation of tumor-suppressor genes, finally leading to dysregulation of cell proliferation, metastasis and drug resistance signals. Non-coding RNAs (ncRNAs) have emerged as one important kind of molecules that can regulate altered genes contributing, to the establishment of metabolic reprogramming. Moreover, diverse metabolic signals can regulate ncRNA expression and activity at genetic, transcriptional, or epigenetic levels. The regulatory landscape of ncRNAs may provide a new approach for understanding and treatment of different types of malignancies. In this review we discuss the regulatory role exerted by ncRNAs on metabolic enzymes and pathways involved in glucose, lipid, and amino acid metabolism. We also review how metabolic stress conditions and tumoral microenvironment influence ncRNA expression and activity. Furthermore, we comment on the therapeutic potential of metabolism-related ncRNAs in cancer.

## Metabolic reprogramming: cancer metabolism changing the energetic state to fulfill cellular requirements

Deregulation of cellular energetics has been pointed out as one of the emerging hallmarks of cancer, both during early cellular transformation and as a driving phenotype of several tumorigenic programs (Kroemer and Pouyssegur, 2008; Munoz-Pinedo et al., 2012). Under physiological conditions, cells maintain regulated and complex metabolic homeostasis by diverse signaling pathways that function as energetic sensors. Metabolic sensors act under a network of cooperative signaling cascades, not only to fulfill the energetic requirements of the cells, but also to influence cellular pathways like cell growth, proliferation, and death (Dumortier et al., 2013). In contrast, cancer cells loose this regulated homeostasis in several ways, including alterations in intrinsic and extrinsic molecular mechanisms that govern cellular metabolism, in order to provide the basic metabolic requirements of tumoral cells, such as quick biosynthesis of ATP, accelerated biosynthesis of macromolecules, and maintenance of optimal redox status (Cairns et al., 2011). To satisfy their metabolic needs, cancer cells also present changes in energetic pathways such as elevated glucose uptake, aerobic glycolysis and altered lipid and fatty-acid metabolism (Newsholme et al., 1985; Vander Heiden et al., 2009). This advantageous bioenergetic state is not only related to the metabolic requirements imposed by the higher biological activity of the tumoral cells, it can also promote a proliferative phenotype and facilitate cell survival and movement under adverse conditions like hypoxia or glucose and nutrient deprivation, becoming a major player in the development and evolution of a tumor (Jones and Thompson, 2009).

This metabolic shift, known as metabolic reprogramming, has been correlated with the activity of oncogenes and loss of tumor suppressor molecules (Esquela-Kerscher and Slack, 2006). Furthermore, once a tumor has developed and reached a certain volume, it becomes difficult to maintain optimal oxygen levels in its cells, creating a hypoxic environment. This also promotes a metabolic reprograming which includes an elevated glycolytic rate, preferentially through oxidative phosphorylation and suppression of gluconeogenesis, creating complex glucose-lactate fluxes, as well as a pro-tumorigenic environment (Reyes et al., 2014).

**Figure 1:**
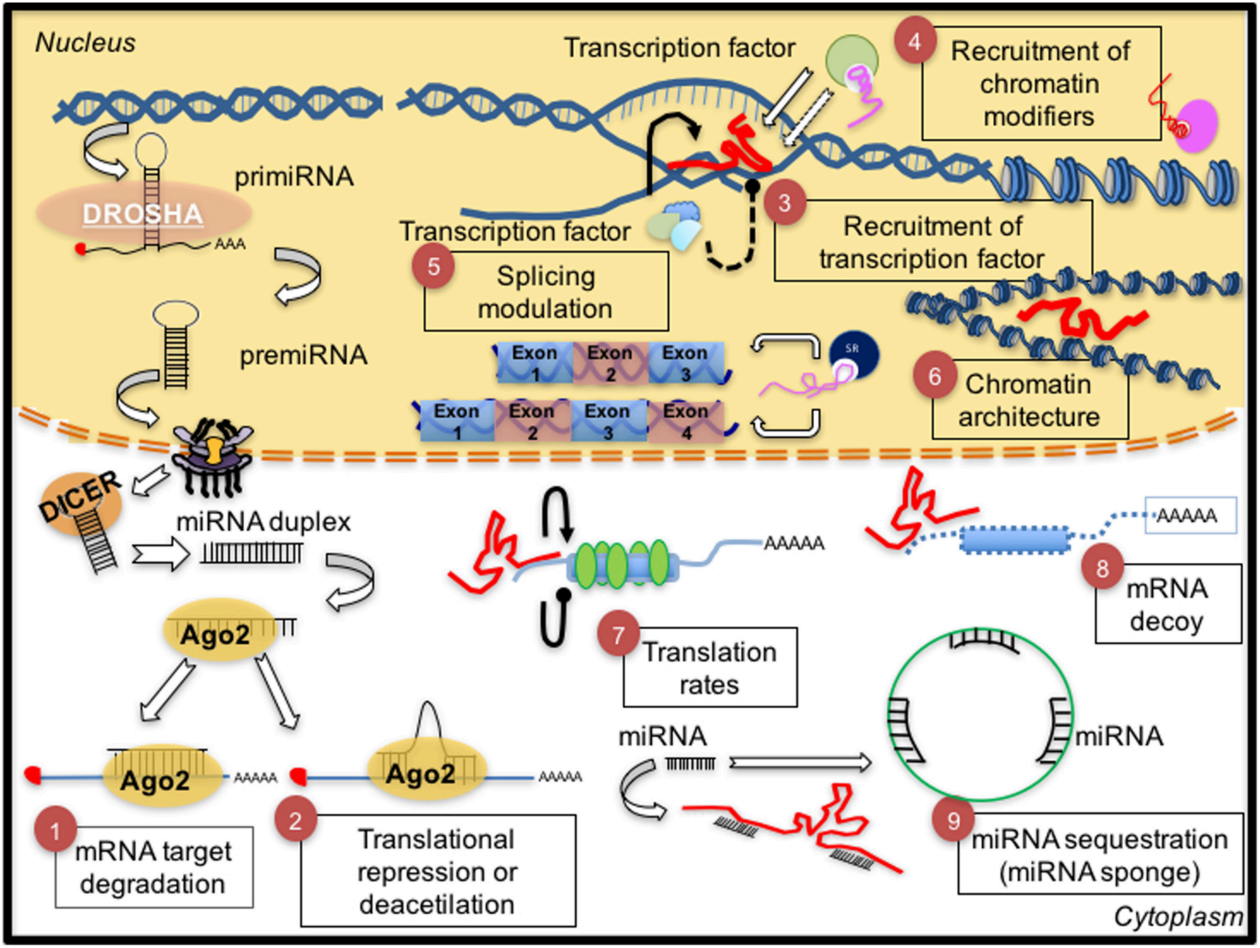
**Biological and mechanical overview of non-coding RNAs**. (1, 2) Biogenesis of microRNAs and their main mechanisms of action. The pri-miRNA is transcribed by pol II polymerase and digested by the RNase DROSHA originating a pre-miRNA (70 nt), which is exported to the cytoplasm by exportin 5. Then, another RNase, Dicer, digests the pre-miRNA to generate a mature duplex miRNA (~22 nt). One strand of this duplex is then incorporated in the miRISC complex (Ago2-microRNA) to target mRNA by perfect complementarity producing transcript degradation (1) or an imperfect one promoting translation repression (2). Conversely, (right side), general functions of lncRNAs are described. (3) Recruitment of transcription factors to promote transcription of target genes or (4) recruitment of chromatin modifiers and thus (6) promoting remodeling of the chromatin architecture. Other functions of lncRNAs are (5) control of alternative splicing of mRNA, and (7) control of translation rates favoring or inhibiting polysome loading to mRNAs, (8) acting as a decoy to preclude access of regulatory proteins to DNA. (9) The interaction between microRNAs and endogenous competent RNAs (ceRNAs) is a redundant system to regulate mRNA expression by lncRNAs-microRNAs; this mechanism is known as sponge function by lncRNAs. Thus, microRNA sequestration by lncRNA prevents microRNA functions on its target.

Non-coding RNAs (ncRNAs), mainly, microRNAs (miRNAs) and long non-coding RNAs (lncRNAs), have been defined as important regulators of several metabolic pathways. miRNAs are small ncRNAs (between 19 and 22 nt), with an important role as post-transcriptional regulators (Bartel, 2009). LncRNAs are transcripts from 200 nt to 100 kilobases (kb) lacking an open reading frame and without evident protein-coding function (Rinn and Chang, 2012). Both of them participate in many physiological processes through the modulation of gene expression at the epigenetic, transcriptional, and post-transcriptional levels (Figure 1).

ncRNAs can actively regulate energetic signaling by targeting key metabolic transporters and enzymes, or by directly or indirectly controlling the expression of tumor suppressors or oncogenes (Iorio and Croce, 2012). Analysis of the correlation between oncogenic programs, metabolic reprograming and aberrant ncRNA expression has highlighted the crucial role of these metabolic aspects in initiation, promotion, and progression of cancer (Arora et al., 2015).

Several lines of evidence suggest that ncRNA plays an important role in the establishment of metabolic reprogramming in cancer cells, as well as the feedback regulation between alterations in energetic signaling and ncRNA expression or activity. In this review, we will discuss the evidence that describes the roles of ncRNAs as modulators of cancer metabolism and as molecules which contribute to the establishment of a diversity of mechanisms that govern the heterogeneity and plasticity of the energetic metabolism of cancer cells.

## ncRNAs regulate glycolytic fluxes: a sweet story

**Figure 2:**
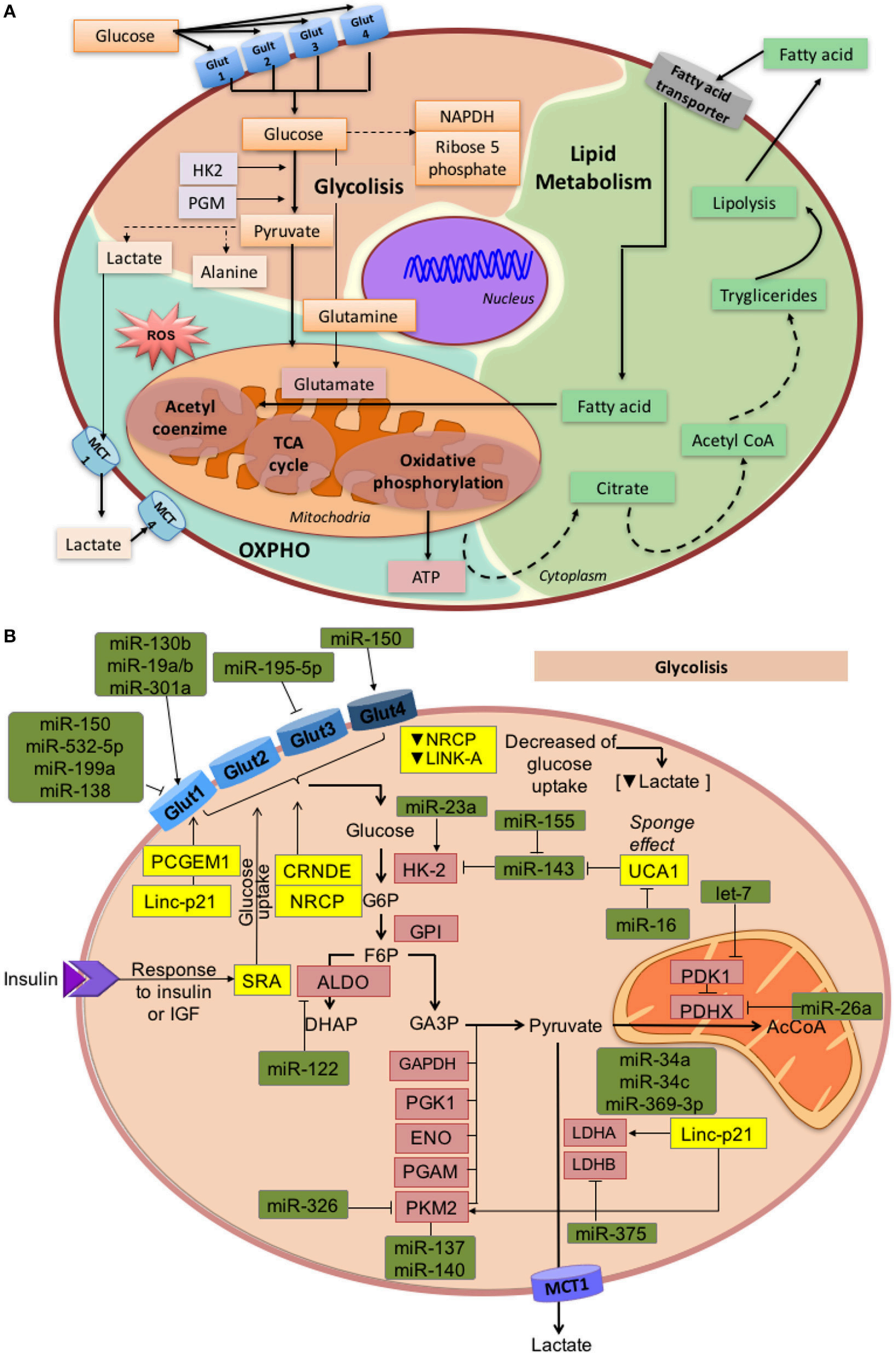
**Overview of glycolysis, OXPHO, and lipid metabolism. (A)** This figure describes the connections between metabolic sub-products that take part in different metabolic process, as well as enzymes and substrates that maintain the normal metabolic environment. Glycolysis occurs in the cytosol when D-glucose is internalized into the cell through the membrane transporters *GLUT1, 2, 3*, and *4*. Through a system of coupled enzymatic reactions, D-glucose is converted into pyruvate, which enters into the TCA cycle, and OXPHO. When the amount of oxygen is limited, pyruvate is converted into lactate. Conversely, in the mitochondria, the TCA cycle is coupled to OXPHO which represents the largest source of metabolic energy. Pyruvate is oxidized and converted into acetyl coenzyme A, which enters the TCA cycle that generates reducing molecules (NADH and FADH_2_) to produce ATP by oxidative phosphorylation. Finally, fatty acids can be converted into acetyl coenzyme A by ß-oxidation to then generate energy through the TCA cycle and OXPHO **(B)**. Glycolysis regulation by miRNAs and lncRNAs under oncogenic conditions. Expression of the GLUT transporter family is regulated by ncRNAs, thus altering the internalization rate of glucose. Other molecules are under ncRNAs regulation pathways, such as hexokinase-2 enzyme, which mediates the transformation of glucose to glucose 6-phosphate, *PKM2* enzyme involved in pyruvate synthesis, *LDHB* and *LDHA* enzymes that convert pyruvate into lactate, and *PDHK*, responsible for the synthesis of Acetyl coenzyme A from pyruvate.

One of the most significant changes induced by cancer metabolic reprogramming involves the bypass of oxidative phosphorylation (Tricarboxylic Acid cycle) to a non-oxidative pathway lead by aerobic glycolysis and lactate production, in order to satisfy the energetic demands of the tumor cells (Vander Heiden et al., 2009). One of the better characterized metabolic phenotypes present in tumor cells is the Warburg effect, which gives preference to ATP generation through glycolysis, even under normal oxygen concentrations, over ATP synthesis through the electron transport chain in the mitochondria (Warburg, 1956; Gatenby and Gillies, 2004; Kim and Dang, 2006). Consequently, most of the glucose in the cell is converted to lactate, rather than being metabolized through the Krebs cycle (Warburg, 1956; Semenza et al., 2001; Gatenby and Gillies, 2004). Although the energetic balance established by glycolysis is less efficient (lower quantity of ATP generated per unit of glucose) than oxidative phosphorylation, it is quicker. However, oxidative phosphorylation is not completely abolished and still functions at a low level (Figure 2A). Therefore, this abnormal and accelerated metabolism meets the energetic needs for cellular functions and construction of biological blocks (fatty acids, lipids, nucleotides, and proteins) for cancer cells (Zheng, 2012).

The first step in energy metabolism is the entry of glucose into the cells through glucose transporters (GLUTs). Until now, 14 isoforms of GLUTs have been identified, of which *GLUT1, 2, 3*, and *4* are well-characterized and expressed in different tissues, some of them in a specific manner (Thorens and Mueckler, 2010). ncRNAs actively regulate the intracellular glucose levels by modulating gene expression of glucose transporters. For instance, *GLUT1* is targeted by miR-340, which is up-regulated in oral squamous cell carcinoma (Xu et al., 2016). In renal cell tumors, miR-199a, miR-138, miR-150, and miR-532-5p down-regulate *GLUT1* expression, whereas miR-130b, miR-19a/b, and miR-301a increase GLUT-1 (Chow et al., 2010). Additionally, loss of miR-1291 enhances the development of renal tumors through targeting *GLUT1* (Yamasaki et al., 2013). In prostate tumors, the PCGEM1 lncRNA promotes the expression of *GLUT1*. Similarly, lncRNA-p21 expression is related to HIF-1α and its responsive genes, such as *GLUT1*, promoting its expression in diverse cancer cell lines (Yang et al., 2014). In bladder cancer, down-modulation of miR-195-5p allows the expression of *GLUT3* (Fei et al., 2012; Peschiaroli et al., 2013). Additionally, reduced levels of miR-150 negatively regulate *GLUT4* expression in pancreatic cancer cells (Srivastava et al., 2011). Such alterations in the expression of ncRNAs and their effect over GLUT expression, represent possible mechanisms through which tumors may bypass regulatory energetic checkpoints by promoting glycolysis, as well as other oncogenic pathways like proliferation, migration, and invasion (Figure 2B).

ncRNAs can also affect the patterns and mechanisms of glucose uptake and glucose/lactate fluxes in cancer cells, promoting aggressive behavior through the establishment of a glycolytic phenotype. The CRNDE (Colorectal Neoplasia Differentially Expressed) lncRNA responds to insulin-like growth factors (IGF) promoting glucose uptake in colorectal cancer (Ellis et al., 2014). Furthermore, the over-expression of the ceruloplasmin lncRNA (NRCP) in ovarian and breast cancer cells, along with the LINK-A lncRNA in triple negative breast cancer, promotes glucose uptake, favoring lactate production and consequently, enhancing tumor progression (Rupaimoole et al., 2015; Lin et al., 2016). In breast tumors, ncRNAs can also function as modifiers of the tumor microenvironment. Under metastatic conditions, tumor cells secret vesicles that carry high levels of miR-122 to non-tumor cells, repressing glucose uptake in the normal cells and facilitating metastasis by increasing nutrient availability for the cancer cells (Fong et al., 2015; Figure 2B).

After glucose uptake, numerous enzymes take part in the catabolism of trioses, pyruvate, and finally lactate. Regulation of glycolytic enzymes by ncRNAs further increases this biological complexity. Hexokinases (*HK*) catalyze ATP-dependent phosphorylation of glucose to glucose-6-phosphate (Robey and Hay, 2006). Interestingly, *HK2* is overexpressed in various tumors and contributes to the establishment of aerobic glycolysis (Mathupala et al., 2009; Vander Heiden et al., 2011). In lung, colon, prostate and head, and neck squamous cell cancers, loss of miR-143 allows *HK2* expression (Fang et al., 2012; Peschiaroli et al., 2013). Similarly, miR-143 locus is deleted in other malignancies (Volinia et al., 2010), and has also been found down-regulated in cervical tumors (Michael et al., 2003; Lui et al., 2007). In bladder cancer cells, miR-155 repress miR-143, allowing up-regulation of *HK2* (Jiang et al., 2012). Moreover, the up-regulation of hipoxia factors suppresses the expression of miR-199a-5p and promotes glycolysis in liver cancer, since the miRNA normally interferes with the expression of *HK2* (Guo et al., 2015). The Urothelial Cancer-Associated 1 lncRNA (UCA1) modulates *HK2* by activation of *STAT3* through the repression of miR-143 (Li Z. et al., 2014). Another member of the hexokinases, *HK1* is also regulated by miR-138 (Peschiaroli et al., 2013). Additionally, in colorectal cancer rosmarinic acid suppress miR-155 repressing the Warburg effect through the mechanism of inactivating the IL-6/STAT3 pathway (Xu et al., 2015).

Another important intermediate step in glycolysis is the conversion of fructose-1,6-bisphosphate to glyceraldehyde 3-phosphate by the aldose enzyme, which is a direct target of miR-122 in liver cells (Fabani and Gait, 2008).

Pyruvate kinase (*PKM*) regulates the final rate-limiting step of glycolysis, which catalyzes the generation of two molecules of pyruvate and two molecules of adenosine triphosphate (ATP; Mazurek, 2011). MiR-124, miR-137, and miR-340 regulate alternative splicing of the PKM gene in colorectal cancer. The switch from isoform *PKM2* to *PKM1* inhibits the glycolysis rate and promotes oxidative phosphorylation (Sun et al., 2012). *PKM2* is also regulated by miR-326 which is down-modulated in glioblastoma cells (Kefas et al., 2010). Furthermore, pyruvate dehydrogenase kinase (*PDHX*) catalyzes the conversion of pyruvate to acetyl coenzyme A and is down-modulated by miR-26a in colorectal cancer, thus impairing mitochondrial metabolism (Chen et al., 2014). Let-7 is a microRNA that is commonly down-regulated in several cancer types. Since *PDK1* is a physiological target of let-7, its down-regulation in tumors facilitates aerobic glycolysis. Furthermore, *PDK1* is critical for Lin28A/B-mediated cancer proliferation, establishing a precise mechanism by which Lin28/let-7 facilitates the Warburg effect to promote cancer progression (Ma et al., 2014; Figure 2B).

Under aerobic glycolysis conditions, oncogenic lesions convert pyruvate to lactate through lactate dehydrogenase (*LDH*) to fulfill their energetic needs (Hatziapostolou et al., 2013). *LDHB* is a target of miR-375, which is down-regulated in esophageal squamous cell and maxillary sinus squamous cell carcinomas (Isozaki et al., 2012; Kinoshita et al., 2012). Another important enzyme is the *LDHA*, frequently overexpressed in tumor cells, and targeted by miR-34a, miR-34c, miR-369-3p, miR-374a, and miR-4524a/b, that are down-modulated in colorectal cancer tissues (Wang J. et al., 2015). Moreover, lncRNA-p21 positively modulates *LDHA*, Enolase 1, *PDHX*, Isozyme 4 (*PDK4*), Phosphoglycerate mutase (*PGAM2*), Glucose-6-Phosphate Isomerase (*GPI*), and Pyruvate Kinase (*PKM2*) in diverse tumors (Hung et al., 2014). The ability of cells to maintain optimal lactate fluxes depends on monocarboxylate transporters (MCTs). Specifically, *MCT1* is targeted by miR-29a, miR-29b, and miR-124 in pancreatic cancer (Pullen et al., 2011). Additionally, let-7b, usually inhibited in tumors, has been shown to target basigin (*BSG*) which interacts with *MCT1* (Fu et al., 2011; Figure 2B).

Cancer cells reprogram their metabolism, based on complex regulatory networks involving diverse oncogenic and tumor suppressor genes, including *PI3K/Akt, Myc*, hypoxia inducible factor (*HIF*), Ras, Src, p53, and PTEN that promote an increase glucose uptake and glycolysis (Dang et al., 2009; Luo and Semenza, 2011). Those genes are targets of ncRNAs regulation networks in cancer (Table 1).

**Table 1:** **ncRNAs and their participation in cancer metabolic processes through oncogenic or tumor suppressor pathways**.

**Pathway**	**ncRNA**	**Biological activity**	**Cancer**	**References**
*PI3K/AKT* signaling: leads to an increase in *HIF*-1α and thus, enhances the expression of glycolytic enzymes (*LDH-B, PKM2, GLUT1*; Zha et al., 2011).	miR-126	Targets the *p85b* subunit of PI3K.	CC, gastric, BRCA.	Guo et al., 2008; Feng et al., 2010; Zhou et al., 2014
	miR-199	Repress *mTOR1* and *c-met*.	HCC	Guo et al., 2008; Fornari et al., 2010
	miR-21	Activates *PI3K/AKT/mTOR* pathway.	BLACA	Yang et al., 2015
	miR-181a	Induces a metabolic shift by inhibiting the expression of *PTEN*, leading to an increase in phosphorylated *Akt*.	CC	Wei et al., 2014
*Akt:* stimulates glycolysis by increasing expression of glucose transporters and glycolytic enzymes	miR-451	Regulates *AMPK* signaling in response to glucose levels by targeting the binding partner of *LKB1, CAB39* (MO25a).	GC	Elstrom et al., 2004; Godlewski et al., 2010
*IGF-I*/insulin signaling: increased expression of genes involved in the regulation of glucose metabolism and mitochondrial function	miR-7	Inhibits cellular growth and glucose metabolism by regulating the *IGF-1R/Akt* signaling pathway.	GC	Wang B. et al., 2014
	miR-126	Negatively regulates *IRS1*, an adaptor protein mediating *IGF-I/insulin* signaling, leading to activation of the *PI3K, Akt* and *Ras-MAPK* pathways.	Mesothelioma, HCC	Ryu et al., 2011; Tomasetti et al., 2014
	miR-33a/b	Controls the expression of Irs2 affecting *Akt* phosphorylation. Also, represses *AMP-activated kinase 1 (Ampkα1)* and *sirtuin 6 (Sirt6)*, involved in the regulation of lipid and glucose metabolism.	BRCA	Davalos et al., 2011
*c-Myc*: The oncogene deregulates glycolysis through the activation of several components of the glucose metabolic pathway.	miR-23	*c-Myc* transcriptionally represses miR-23a/b, which targets glutaminase (*GLS*) inducing mitochondrial dysfunction.	Lymphoma and PCA	Gao et al., 2009
	lncRNA PCGEM1	Stimulates the uptake of glucose by aerobic glycolysis and interacts directly with *c-Myc*, and enhances its transactivation activity by its recruitment to chromatin.	PCA	Dang et al., 2009; Hung et al., 2014
*HIF* signaling: key transcription factor mediating responses to hypoxia, and *HIF*-target genes, implicated in deregulated tumor metabolism.	miR-199a and miR-125b	Directly targets *HIF-1α* and other miRNAs, enhancing tumor angiogenesis.	OC	He et al., 2013
	miR-424	Hypoxia-inducible miRNA, that targets cullin (*CUL2*), which stabilizes *HIF-1α*.	OC (endothelial cells)	Ghosh et al., 2010
	miR-17-92	Down-regulates *HIF-1α*, leading to evasion of apoptosis.	LC	Taguchi et al., 2008
	miR-451	Reduces activation of the *LKB1/AMPK* pathway, facilitating unrestrained *mTOR* activity.	GB	Godlewski et al., 2010
*P53*: Its down-modulation provides a selective advantage for cancer cells by increasing glycolysis.	miR-34	Loss of its expression interrupts *p53*/miR34 feedback resulting in lower activity of both molecular actors, leading to the over-expression of glycolytic enzymes (*HK1/2, GPI*, and *PDH1*).	Most tumors	Voorhoeve et al., 2007; Kumar et al., 2011; Kim et al., 2013
	miR-25, 30d, 504, and 125b	Directly target *p53* and impairs p53 response.	Gastric, brain and LC	
	miR-372 and 373.	Neutralizes *p53*-mediated *CDK* inhibition, by silencing LATS2.	Testicular germ cell tumors	
	lncRNA MEG3	Down-modulation of *MEG3* disturbs the activation of *MDM2* and *p53*.	Non-small cell LC	Lu et al., 2013

*LC, lung cancer; HCC, hepatocellular carcinoma; BlaCa, Bladder cancer; CC, colon cancer; BRCA, breast cancer; PCA, prostate cancer; GC, glioblastoma cancer*.

Not only can the human genome-encoded ncRNAs modulate glucose metabolism in cancer cells. Kaposi's sarcoma-associated herpesvirus (KSHV), the etiological agent of Kaposi's sarcoma, has been shown to express microRNAs in its genome that collaborate to induce aerobic glycolysis in infected cells, mainly through the down-regulation of *EGLN2* and *HSPA9*, which cooperate to form the glycolytic phenotype (Yogev et al., 2014).

## Lipid metabolism: a fat story

**Figure 3:**
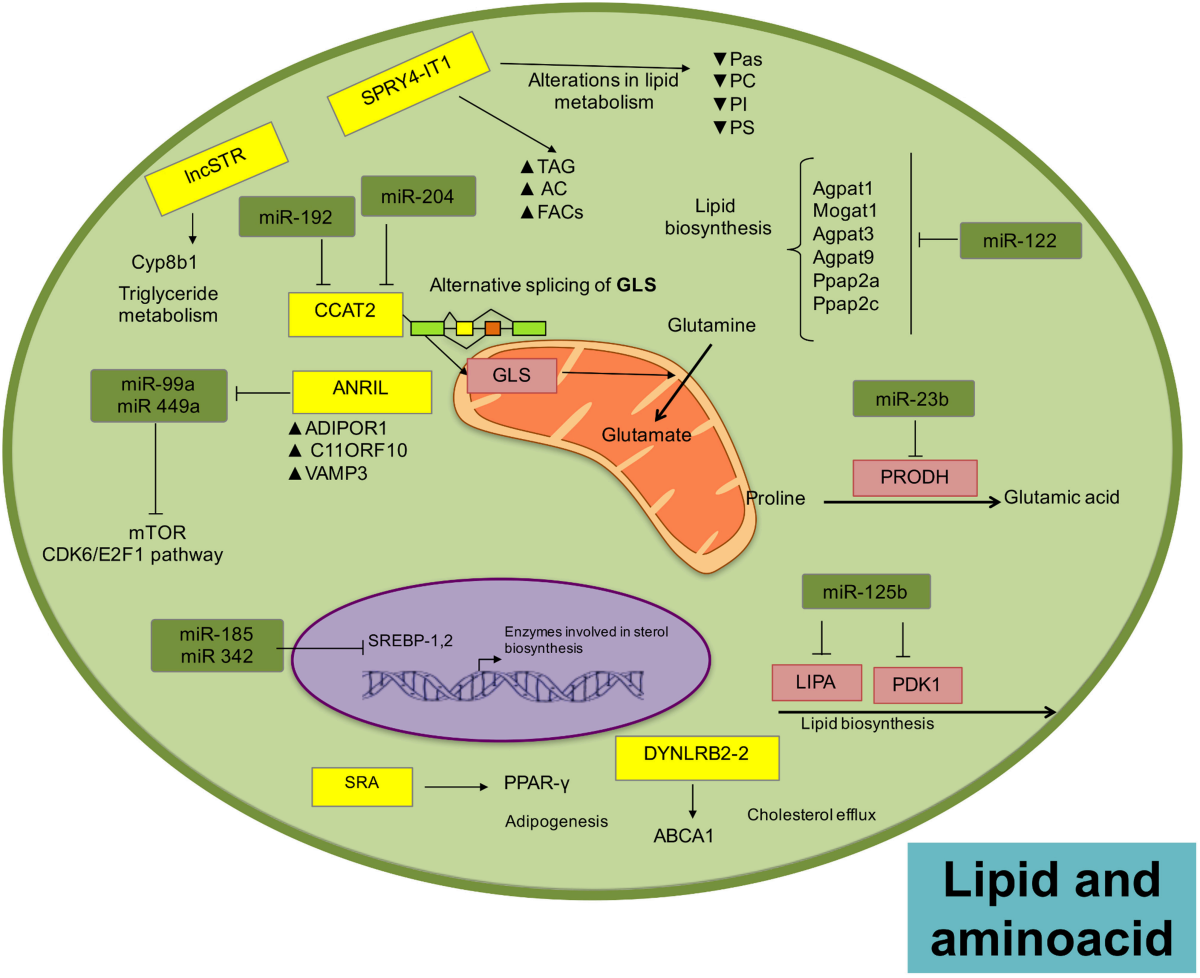
**Landscape of lipid and amino acid metabolism regulated by ncRNAs in tumors**. Micro and lncRNAs can regulate the metabolism of amino acids through the regulation of enzymes related with these metabolic pathways, favoring the disposition of amino acids as important sources of energy. There is also a fine regulatory loop between microRNAs and lncRNAs than can actively impact metabolic networks.

Lipids constitute a mayor building block for organelles and cells to maintain cellular function and structure provide energy and orchestrate different cellular pathways. As part of lipid metabolism (anabolism and catabolism) a variety of biological intermediators are generated as second messengers (Huang and Freter, 2015), which manage multiple signaling pathways like cell growth, proliferation, differentiation, survival, apoptosis, inflammation, motility, and membrane homeostasis (Mattes, 2005; Krycer et al., 2010; Zechner et al., 2012). Alterations in lipid metabolism can affect cell function, promoting the establishment, and development of cancer (Santos and Schulze, 2012). In fact, lipid biosynthesis is limited to a subgroup of tissues and organs, including adipose, liver, and breast, but its reactivation or reprogramming is commonly observed in tumor cells (Menendez and Lupu, 2007; Abramson, 2011; Beloribi-Djefaflia et al., 2016). The activation or inhibition of lipid signaling pathways is aimed at fulfilling the cell energy requirements and responds to environmental changes. There are numerous enzymes regulating lipid metabolism in the cells and recently, diverse data show that expression of many of these enzymes are regulated by ncRNAs (Huang and Freter, 2015; Figure 3).

In prostate cancer cells, miR-185 and miR-342 control lipogenesis and cholesterol synthesis by down-modulating the expression of sterol regulatory element binding protein 1 and 2 (*SREBP-1, SREBP-2*), repressing their responsive genes, including fatty acid synthase (*FASN*) and 3-hydroxy-3-methylglutaryl CoA reductase (*HMGCR*; Li X. et al., 2013). In lymphocytic leukemias, metabolic enzymes related with lipid biosynthesis, such as lipase A (*LIPA*) and pyruvate dehydrogenase lipoamide kinase isozyme 1 (*PDK1*), are targets of miR-125b (Tili et al., 2012). Recently, miR-205 has been associated with lipid metabolism de-regulation in hepatocellular carcinoma, acting on acyl-CoA synthetase long-chain family member 1 (*ACSL1*), a lipid metabolism enzyme in liver (Liu et al., 2012; Cui M. et al., 2014). Additionally, the loss of miR-122, an abundant liver-specific miRNA, alters fat and cholesterol metabolism through modulation of genes involved in lipid synthesis, including *Agpat1, Mogat1, Agpat3, Agpat9, Ppap2a, Ppap2c* (Hsu et al., 2012; Tsai et al., 2012).

Over-expression of miR-27a in hepatitis C virus-infected liver cells vs. hepatitis B virus-infected cells has been recently described. MiR-27a targets the lipid synthetic transcription factor *RXR* and the lipid transporter ATP-binding cassette subfamily A member 1 in hepatocarcinoma. Moreover, miR-27a down-modulates the expression of several lipid metabolism-related genes (*FASN, SREBP1, SREBP2, PPAR, PPAR, ApoA1, ApoB100, and ApoE3*), some of which also participate in the production of infectious viral particles (Shirasaki et al., 2013).

The over-expression of *HULC* contributes to the malignant development of hepatocellular carcinoma by supporting abnormal lipid metabolism via activation of the acyl-CoA synthetase subunit *ACSL1*. This results in promotion of lipogenesis and the accumulation of intracellular triglycerides and cholesterol in experimental models. HULC induces methylation of the miR-9 promoter, regulating its expression and favoring alterations in lipid metabolism (Cui et al., 2015). LncRNA SPRY4-IT1 was first identified in adipose tissue (Ota et al., 2004) and was recently found up-regulated in melanoma (Khaitan et al., 2011). Expression of this lncRNA shows a strong correlation with lipid metabolism alterations, including the increase of acyl carnitine, fatty acyl chains, and triacylglycerol, as well as the down-modulation of phosphatidic acid, phosphatidylcholine, phosphatidylinositol, and phosphatidylserine. It is probable that the significant changes in lipid profiles are correlated with the oncogenic modulation of SPRY4-IT1 over the lipid phosphatase lipin 2, an enzyme that converts phosphatidate to diacylglycerol (Mazar et al., 2014).

The oncogene ANRIL is up-regulated in gastric, lung, hepatocellular, cervical, melanoma, ovarian, bladder cancer, among other tumors (Li Z. et al., 2016). Interestingly, ANRIL regulates genes involved in glucose and fatty acid metabolism (Bochenek et al., 2013), such as *ADIPOR1*. Furthermore, ANRIL can epigenetically regulate the expression of miRNAs in gastric cancer cells, particularly miR-99a/miR-449a, which target *CDK6/E2F1* and *mTOR* pathways (Zhang et al., 2014), that regulate lipid metabolism and adipose tissue function (Cai et al., 2015).

The steroid receptor RNA activator gene is an unusual gene that expresses two different transcripts by alternative splicing of the first intron: (1) the lncRNA SRA and (2) the SRAP protein gene (Hube et al., 2006). SRA co-activates *PPAR-gamma*, inducing adipogenesis (Xu et al., 2010; Liu et al., 2014) and it may also regulate lipid metabolism (Marion-Letellier et al., 2015). Interestingly, the over-expression of SRA has been associated with poor prognosis in endometrial cancer (Smolle et al., 2015). The lncRNA-DYNLRB2-2 responds to oxidized-LDL to promote *ABCA1*-mediated cholesterol efflux (Hu et al., 2014). In prostate cancer, the ox-LDL/lncRNA-DYNLRB2-2 circuit might be involved in the promotion of proliferation, migration and invasion rates (Wan et al., 2015). Experiments in animal models showed that the lncLSTR (lncRNA-liver-specific triglyceride regulator), a liver-enriched lncRNA, physiologically contributes to triglyceride metabolism by enhancing *Cyp8b1* expression, a molecule involved in bile acid synthesis. Furthermore, *Cyp8b1* is down-modulated in primary hepatocytes in which lncLSTR is depleted, suggesting a regulatory activity over *Cy8b1* as one of its downstream responsive genes (Li et al., 2015a).

## Amino acid metabolism

Apart from other energetic sources, amino acids are important substrates that sustain mitochondrial metabolism and support the biosynthesis of proteins, lipids, and other macromolecules. Alterations in amino acid metabolism are also common in cancer cells (Figure 3).

Glutamine metabolism seems to have a critical role in cancer programs, and has been implicated in tumor formation and metastasis (DeBerardinis and Cheng, 2010), as well as being an important source of tumor energy (Li and Zhang, 2016). miRNAs have also been reported to regulate amino acid catabolism, for example, in kidney cancer miR-23b^*^ regulates proline oxidase, which is the first enzyme involved in the conversion of proline to glutamic acid (Liu et al., 2012). Interestingly, the lncRNA CCAT2 participates in the alternative splicing of glutaminase (*GLS*), an enzyme that catalyzes the hydrolysis of glutamine to glutamate (Redis et al., 2016), where glutamate can be further deaminated to a-Ketoglutarate by glutamate dehydrogenase (*GDH*) and incorporated into the tricarboxylic acid cycle (Li and Zhang, 2016). Another lncRNA involved in glutamine metabolism is PCGEM1 an androgen-induced prostate specific lncRNA, which regulates expression of enzymes such as GLS, Glutathione Reductase (*GSR*), and type I gamma-glutamyltransferase (*GGT1*) in prostate tumors (Hung et al., 2014).

Redundant regulation by ncRNAs reveals that metabolic pathways in cancer are finely regulated, acting at different cellular levels. Consequently, understanding these processes will enable future development of anti-metabolite therapies to target specific energetic signals altered in oncogenic lesions.

## Mitochondrial metabolism in cancer and its relation with ncRNA

**Figure 4:**
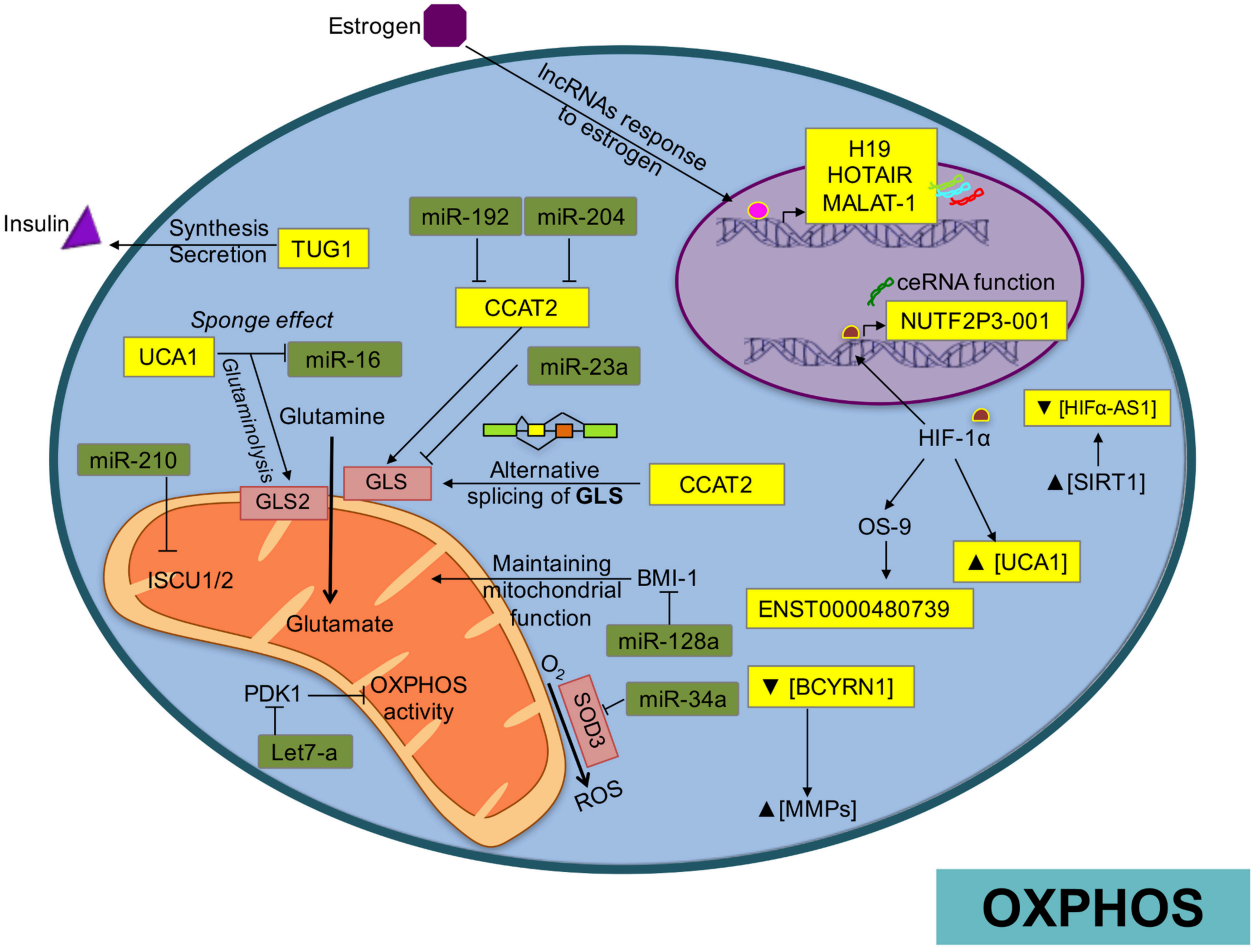
**Overview of ncRNA regulatory network over OXPHO in cancer**. In this picture we show how ncRNAs are involved in the regulation of OXPHOS, generation of ROS, or mediating alternative splicing of mitochondrial enzymes.

The partial maintenance of mitochondrial function in glycolytic cells appears essential for cancer cell development. Thus, the tumor must balance the bioenergetic requirements to grow, proliferate, and survive within the energetic restrictions and metabolic pathways. Mitochondria are in fact, the main intracellular producers of reactive oxygen species (ROS) as part of adenosine triphosphate (ATP) production through oxidative phosphorylation (OXPHOS). This organelle is responsible for converting available nutrients into the fundamental blocks required for cell maintenance (Ahn and Metallo, 2015), such as fatty acids, cholesterol and proteins (Kamphorst et al., 2013). Therefore, mitochondrial alterations have been implicated in the etiology of many diseases including cancer. The metabolic reprogramming of the mitochondrial network in tumoral programs is achieved through several mechanisms, including ncRNAs transcribed both in the nuclear and in the mitochondrial genome (mtDNA). ncRNAs can actively regulate mitochondrial metabolism by controlling structural and functional mechanisms that respond to changes in energy requirements or environmental conditions (Benard et al., 2010; Figure 4).

For example, miR-210 is up-regulated by hypoxia (Dang and Myers, 2015), and can block mitochondrial respiration through down-modulation of the electron transport chain (ETC) complexes (Huang and Zuo, 2014). Particularly, miR-210 targets *ISCU1* and *ISCU2*, suppressing mitochondrial function and disrupting iron homeostasis in colon, breast, and esophageal cancer (Chen et al., 2010; Favaro et al., 2010). In breast cancer cells, miR-378^*^ promotes a metabolic shift by inhibiting the expression of important regulators of energy metabolism such as estrogen-related receptor-γ and GA-binding protein transcription factor. This reduces the tricarboxylic acid cycle (TCA) rates, decreasing the dependency on OXPHOS, and increasing lactate production (Eichner et al., 2010). Similarly, in hepatocellular carcinomas miR-23a modulates a metabolic switch from OXPHOS to anaerobic glycolysis by targeting the glucose-6-phosphatase catalytic subunit (*G6PC*), which plays an important role in mitochondrial respiration (Wu et al., 1999; Wang et al., 2012). Likewise, overexpression of miR-125b in lymphocytic leukemia models represses many transcripts implicated in energetic and lipid metabolism including phosphatidylcholine transfer protein, lipase A, lysosomal acid, cholesterol esterase, glutathione synthetase, acyl-CoA synthetase short-chain family member 1, *HK2*, stearoyl-CoA desaturase 1, *AKT2*, and pyruvate dehydrogenase kinase 1 (*PDK1*; Tili et al., 2012).

Some of the most important by-products of the electron transport chain in the mitochondria are reactive oxygen species (mROS). Increased production of ROS can lead to activation of tumorigenic signaling and metabolic reprogramming. This tumorigenic signaling includes mechanisms to prevent imbalances in the production of mROS maintaining redox homeostasis (Sullivan and Chandel, 2014). Emerging evidence shows that control of ROS levels is mediated in part by ncRNAs. One of the first evidence was the cluster miR-17–92, overexpressed in small-cell lung cancer, which reduce DNA damage to a tolerable level and consequently lead to the accumulation of genetic instability (Ebi et al., 2009). miR-141 and miR-200a, contribute to ovarian tumorigenesis by targeting *p38*α and modulating oxidative stress response in mouse models (Mateescu et al., 2011). In addition, miR-21 and miR-34a promote tumor malignancy by the formation of ROS through the mediation of *SOD3* and *TNF*α expression in cancer cells (Zhang et al., 2012). In medulloblastoma, miR-128a regulates ROS by specific inhibition of the Bmi-1 oncogene, which participates in maintaining mitochondrial function and redox homeostasis (Venkataraman et al., 2010). Let-7a promotes OXPHOS in breast cancer (Serguienko et al., 2015) and hepatocellular carcinoma by directly modulating *PDK1*, which as mentioned previously, is a negative regulator of OXPHOS activity (Ma et al., 2014). In bladder cancer, the lncRNA UCA1 participates in ROS formation and promotes mitochondrial glutaminolysis by its sponge effect on miR-16 (Li H. J. et al., 2015). *SOD2*, which has response elements for *NF*-κ*B*, wipes out the superoxide anion radicals generated by OXPHOS and coverts them into hydrogen peroxide in cancer cells. Although it is know that the lncRNA Lethe prevents binding of *NF*κ*B* to *NF*κ*B* response elements resulting in the suppression of SOD2 (Rapicavoli et al., 2013), the impact of Lethe on energetic metabolism of cancer cells is poorly understood.

Apart from glucose, cancer cells exhibit increased glutamine intake and glutamine metabolism (glutaminolysis). The accelerated glutamine metabolism provides enough substrate to increase lipogenesis and nucleic acid biosynthesis, necessary for the proliferative phenotype of the cancer cells (Gao et al., 2009). Of particular importance, mitochondrial enzymes participate in the metabolism of glutamine and other metabolites (glutamate, proline, aspartate, and alanine) as part of the tumor programs (Dang, 2010). One of the major regulators of glutaminolysis is *MYC*. Along the same line the suppression of miR-23A/B by *MYC* enhances mitochondrial glutaminase expression and glutamine metabolism in prostate cancer (Gao et al., 2009). Additionally, the deregulation of the HOTTIP lncRNA by miR-192 and miR-204 can produce an abnormal glutaminolysis through positive regulation of *GLS1* in hepatocellular tumors (Ge et al., 2015). Furthermore, the CCAT2 lncRNA modulates GSL alternative splicing through an allele-specific regulatory mechanism (Redis et al., 2016). Moreover, in bladder cancer cells the UCA1 lncRNA promotes glutamine metabolism through its sponge function over miR-16, allowing the expression of *GLS2*, enzyme that participates in the hydrolysis of glutamine to glutamate (Li H. J. et al., 2015).

The involvement of mitochondrial miRNAs (mitomiRs) and mitochondrial lncRNAs in regulating the OXPHOS system is of particular interest. These regulatory molecules have either a pro-oxidant or antioxidant effect (Bai et al., 2011; Aschrafi et al., 2012; Li P. et al., 2012). Therefore, mitochondrial ncRNAs may participate in the fine-tuning of the mitochondrial energy supply. A recent study identified 13 miRNAs significantly enriched in mitochondria of HeLa cells, which actively participate in cell cycle and apoptosis through regulation of mitochondrial activity (Bandiera et al., 2011; Demongeot et al., 2013). The lncRNAs encoded by mtDNA, ASncmtRNA-1/2, are down-regulated in cancer cells and take part in the mitochondrial reprograming of oncogenic pathways (Burzio et al., 2009). Biological activity of ASncmtRNAs results in survivin inhibition at the RNA level, probably mediated by microRNAs (Vidaurre et al., 2014). Survivin enhances the stability of oxidative phosphorylation complex II, which promotes cellular respiration (Rivadeneira et al., 2015).

Another type of non-coding RNA, the Plement-induced wimpy testis (PIWI)-interacting RNAs (piRNAs), have been recently recognized to be relevant in cancer metabolic reprogramming. piRNAs are small non-coding RNAs (26–31 nt) that form the piRNA-induced silencing complex (piRISC). The main function of piRNAs is to silence transposable elements (TEs) in the germ line, but also in cancer cells (Siomi et al., 2011), mainly through epigenetic regulation, genome re-arrangement, and stem cell self-renewal (Ross et al., 2014). piRNA expression has been detected in mitochondrial RNAs of HeLa cells, and are possibly implicated in diverse functions related to energetic homeostasis, bioenergetics and cell growth (Kwon et al., 2014).

## Interplay between ncRNAs, tumor microenvironment, and metabolic conditions

Novel data suggest that the regulatory role of ncRNAs during carcinogenesis is not limited to cancer cells they are also implicated in the activation of the tumor stroma and in its transition into a cancer-associated microenvironment. In fact, tumor development involves a fine interplay between malignant and stromal cells. Secreted ncRNAs can serve as regulatory signals promoting cancer cell proliferation, migration, communication, and stromal modification, thereby enhancing an optimal microenvironment for oncogenesis (Soon and Kiaris, 2013). The tumor microenvironment presents a complex architecture, comprising fibroblasts, vascular endothelial cells, immune cells, adipocytes, and extracellular matrix, conforming the stromal tissue that surrounds and interacts tumor cells (Hanahan and Weinberg, 2011).

Importantly, cancer-associated fibroblasts (CAFs) can modify the metabolism of the adjacent cancer cells, as a consequence, its activity can promote tumor growth, invasion and angiogenesis (Franco et al., 2010). CAFs are originated from normal fibroblasts (NFs) that are in contact with tumor cells, receiving and sending signals to co-evolve with the tumor cells and support their biological requirements. Communication pathways between CAFs and neoplastic cells include ncRNA mediated signaling (Table 2; Erez et al., 2010).

**Table 2:** **ncRNAs and tumor microenvironment**.

**ncRNA**	**Microenvironment component and its activity**	**References**
**CANCER-ASSOCIATED FIBROBLASTS (CAFs)**		
miR-149	Inhibits fibroblast activation by targeting *IL-6*. It is suppressed in gastric cancer.	Li et al., 2015b
miR-424	Regulates *IDH3α* expression in melanoma and colon cancer cell line models triggering the metabolic switch from oxidative phosphorylation to glycolysis in CAFs.	Zhang et al., 2015
miR-133b	In prostate cancer (PCA), its overexpression modulates *IL6*-activation, and other miRNAs, including miR-210, miR-143, and miR-590-5p, that coherently up-modulate CAF activation. miR-133b is also released into the media and its incorporation into PCa cells, may contribute to the establishment of mesenchymal phenotype.	Doldi et al., 2015
ZEB2NAT	In bladder cancer, CAFs induces EMT and invasion through the *TGFβ1-ZEB2NAT-ZEB2* axis.	Zhuang et al., 2015
**IMMUNOLOGICAL ENVIRONMENT**		
miR-21	It suppresses antitumor T-cell-mediated immunity and density in colorectal carcinoma.	Mima et al., 2016
miR-142	Regulates proliferative responses and maturation of T cell cycling by mediating *E2F* transcription factors (Sun Y. et al., 2015). In hepatic and colon cancer, miR-142 is down modulated, while in breast cancer it is over-expressed.	Shen et al., 2013; Chai et al., 2014; Isobe et al., 2014
miR-101 and 26a	In ovarian tumors, the overexpression of the miRNAs imposed glucose restriction on T cells, limiting the expression of the methyltransferase *EZH2*.	Zhao et al., 2016
lnc-DILC	*IL-6* autocrine signal in hepatome depends on lnc-DILC and consequently, its expression enhances the activation of *IL-6/STAT3* pathway.	Wang et al., 2016
**ADIPOCYTES**		
miR-27a	Its excretion from adipose tissue leads liver cancer cells to proliferate through the down-regulation of the transcription factor *FOXO1*. *FOXO1* in particular, plays a significant role in regulating energy metabolism and gluconeogenic enzymes (Gross et al., 2008).	Sun B. et al., 2015
miR-143	Its down-modulation promotes adipocyte differentiation in cancer cell lines. Its expression level may be a cause or a consequence of the undifferentiated state of the tumor cells.	Esau et al., 2004
lncRNA SRA	It responds to insulin, and its altered expression in tumor cells may allow both glucose uptake and phosphorylation of *Akt* and *FOXO1* in adipocytes.	Xu et al., 2010

Additionally, the metabolic status in cancer lesions is also balanced by different micro-environmental components. For instance, surrounding immune cells present active alternative pathways to overcome tumor energetic limitations. In particular, the metabolic switch in tumor cells promotes the presence of tumor-infiltrating lymphocytes (T cells) which is a crucial tumoral adaptation to dampen antitumor immunity (Molon et al., 2016; Zhao et al., 2016). Maintaining tumor metabolic homeostasis requires a balanced immune response, which is achieved by extracellular signals that can be induced or repressed by ncRNA activity (Table 2; Dumortier et al., 2013).

Another important component of the tumor microenvironment are the adipocytes, that are considered as an energy storage depot, as well as endocrine cells that produce hormones, growth factors, cytokines, and adipokines (Rajala and Scherer, 2003). Therefore, mature adipocytes influence tumor behavior through heterotypic signaling processes, providing fatty acids for rapid tumor growth, and also promoting homing, migration, and invasion of tumor cells (Nieman et al., 2011). ncRNAs can actively participate as important modulators of the lipid metabolism in tumors where adipocytes represent the major component of the tumoral microenvironment (Table 2).

Emerging data suggest a fine regulatory loop between the HIF system, microenvironment and tumor cells, governed by diverse regulatory molecules like ncRNAs. Given the fact that HIF target genes include many metabolism-induced genes, such as ncRNAS (Semenza, 2010; Masson and Ratcliffe, 2014), and both tumor and stromal hypoxia, along with deregulated metabolism, characterize aggressive cancer phenotypes, it is tempting to conclude that activation and regulation of *HIF* pathways by complex signaling processes is one of the most important causes for deregulated tumor metabolism (Höckel and Vaupel, 2001; Table 3). A more detailed overview about hypoxia and lncRNA is discussed in Chang et al. (2016).

**Table 3:** **ncRNA regulation by hypoxia and hormone environment**.

**ncRNA**	**Activity**	**References**
**HYPOXIA: HYPOXIA FACTORS REGULATED BY ncRNAs**		
miR-17-92 cluster, 107, 20b and 22	They modulate tumor growth by inhibiting *HIF-1α* expression in cancer models.	Yamakuchi et al., 2011
miR-519c	Its overexpression reduced *HIF-1α* levels, followed by tumor angiogenesis, growth, and metastasis suppression.	Cha et al., 2010
miR-138	Directly targets *HIF-1α*, reversing HIF-1α-mediated induction of ovarian cancer cell invasion.	Ye et al., 2014
miR-33a	*HIF-1α* is a direct target in melanoma, where miR-33a has a lower expression and could promote cell proliferation, invasion, and metastasis.	Zhou et al., 2015
ENST00000480739	Its down-modulation abolished pancreatic ductal adenocarcinoma cell invasion and metastasis by indirectly targeting *HIF-1α*.	Sun et al., 2014
**HYPOXIA: ncRNAs REGULATED BY HYPOXIA**		
miR-210, 193b, 145, 125-3p, 708, and 517a	Induced by hypoxic conditions in bladder cancer. Particularly, miR-145 is a direct target of *HIF-1α* (it presents 2 hypoxia response elements in its promoter) and its up-regulation contributes to increased apoptosis.	Blick et al., 2015
miR-124 and miR-144	Hypoxia induced miRNAs, their expression may contribute to a pro-survival mechanism of prostate cancer cells to hypoxia and irradiation.	Gu et al., 2016
Circulating exosomal miR-21	Its expression level is associated with *HIF-1α/HIF-2α* expression, T stage, and lymph node metastasis in oral squamous cell carcinoma. The hypoxic microenvironment may stimulate tumor cells to generate miR-21-rich exosomes that are delivered to normoxic cells to promote prometastatic behaviors.	Li L. et al., 2016
miR-338-3p	Targeted by *HIF-1α* in nasopharyngeal cancer, acting in the initiation and progression of the tumor.	Shan et al., 2015
UCA1	Up-regulated by *HIF-1* facilitating proliferation, migration, invasion, and apoptosis resistance in bladder cancer cells.	Xue et al., 2014
lnRNA-LET	Its down-regulated expression was associated with metastasis in hepatocellular carcinoma (HCC).	Yang et al., 2013
lincRNA-p21	Takes part in a positive feedback loop to stabilize hypoxia-induced *HIF-1α* expression. lncRNA-p21 excludes the binding of *HIF-1α* to *VHL* (an ubiquitin E3 ligase) in prostate cancer.	Yang et al., 2014
AK058003	Frequently up-regulated in gastric cancer as a hypoxia-induced gene, which promotes migration and invasion *in vivo* and *in vitro*.	Wang Y. et al., 2014
lncRNA-NUTF2P3-001	Over-expressed in pancreatic cancer cells under hypoxia. NUTF2P3-001 regulates *KRAS* expression through competing endogenous RNA (ceRNA) function with miR-3923 contributing to oncogenesis.	Li X. et al., 2016
NEAT1	In breast cancer cells, hypoxia induces its expression by enhancing the establishment of active histone marks.	Choudhry et al., 2015
**HORMONES**		
H19, HOTAIR, and MALAT-1	Inducible lncRNAs of estrogens or estradiol in breast cancer.	Zhao et al., 2014; Sun H. et al., 2015; Bhan and Mandal, 2016
NEAT1	In estrogen receptor-positive breast cancer showed greater expression compared to the non-positive tumors.	Choudhry et al., 2015
miR-378^*^	Regulated by *Erb-B2* receptor tyrosine kinase 2 and insulin, induce a metabolic shift in breast cancer cells.	Eichner et al., 2010
miR-135b	Direct regulator of androgen receptor levels in prostate cancer. Its expression is lower in *ERα*-positive breast tumors vs. *ERα*-negative samples, since *ERα* is a direct target of the miRNA. miR-135b also inhibits the *HIF1α*.	Aakula et al., 2015
miR-32, 148a, 99a, 21, and 221	Showed an enrichment in ChIP-seq data of *AR*-binding sites in androgen-responsive prostate cancer LNCaP cells.	Jalava et al., 2012

Endogenous and exogenous hormone-signaling pathways serve as metabolic regulatory networks that control fuel and energy metabolism on both tumor and stromal cells, and connects nutrient availability with cell growth and proliferation. Currently, ncRNA modulation by hormones can reenforce hormone-signaling activity. For example, insulin, a major hormone in the homeostasis of energy and metabolism, has been implicated in the regulation of miRNA expression (Granjon et al., 2009). Additionally, the estrogen receptor activates autophagic fluxes as a response to metabolic damage, in part by regulating ncRNA expression (Bernales et al., 2007; Table 3).

## nc-RNAs mediating metabolic stress responses: autophagy, EMT, angiogenesis, and inflammation

When metabolic stress triggers energetic and nutritional changes in tumor cells, the metabolic stress responses collaborate to maintain homeostasis. Metabolic changes take place in reaction to stress in the tumor and stromal cells through the activation of several mechanisms, including autophagy, epithelial-mesenchymal transition (EMT), angiogenesis, and inflammation.

Autophagy is a catabolic process indispensable for the maintenance of cellular homeostasis. Alterations of autophagy are described in cancer and are due to alterations in the expression of various genes that promote or suppress it (Lozy and Karantza, 2012). Autophagic programs consist of the degradation of cellular organelles, cytoplasmic proteins and lipids, allowing recycling of the resulting catabolites for biosynthesis and energy metabolism, in order to satisfy nutrient, energy and hormonal demands of the tumor cells (Jing et al., 2015). The metabolic requirements of cancer cells are maintained, in part, by autophagy pathways present in tumor or stroma cells (Martinez-Outschoorn et al., 2011; Mathew and White, 2011). Considering the vast implications if ncRNAs in stress responses, their activity might contribute to the dynamics of autophagy during cancer progression (Leung and Sharp, 2010; Table 4).

**Table 4:** **ncRNAs and their contribution to events in the metabolic changes in cancer**.

**ncRNA**	**Activity/Target**	**Cancer type**	**References**
**AUTOPHAGY**			
miR-30a	Autophagy induction/*BECN1*	CML	Yu et al., 2012
miR-17	Vesicle nucleation and elongation/*BECN1* and *ATG7*	Lung, GBM	Comincini et al., 2013; Chatterjee et al., 2014
miR-101	Vesicle elongation/*ATG4*	BRCA	Frankel et al., 2011
miR-204	Vesicle elongation/*LC3*	RCC	Mikhaylova et al., 2012
miR-375	Vesicle elongation/*ATG7*	Hepatic	Chang et al., 2012
miR-23b	Vesicle elongation/*ATG12*	Pancreatic	Wang et al., 2013
miR-130a	Retrieval fusion/*ATG2B*	CLL	Kovaleva et al., 2012
miR-34a	Retrieval fusion/*ATG9*	BRCA	Li L. et al., 2013
miR-182	*BCL-2*	Melanoma	Yan et al., 2012
miR-210	*BCL-2*	Neuroblastoma	Chio et al., 2013
miR-100	*mTOR* pathway genes	Hepatic	Ge et al., 2014
miR-224b	The miRNA is removed by the autophagosome-lysosome pathway	Hepatic	Lan et al., 2014
lncRNA MEG3	Suppressed autophagy activation	Bladder	Ying et al., 2013
**ANGIOGENESIS**			
miR-382↑, 21↑, 17–92↓, 467↑	Pro-angiogenic: *PTEN, RhoB, TSP-1*	GC, PCA, OvCa, BRCA	Fish et al., 2008; Ramøn et al., 2011; Bhattacharyya et al., 2012; Seok et al., 2014
miR-218↓, 18a↑, 145↓, 22↓, 107	Anti-angiogenic: *PLC_γ_1/ARAF, mTOR, p70S6K1, HIF-1α, HIF-1β*	GBM, GC, CCC	Yamakuchi et al., 2010, 2011; Zheng et al., 2013; Mathew et al., 2014
MVIH	Inhibited activation of angiogenesis phosphoglycerate kinase 1 (*PKK1*)		Yuan et al., 2012
**EMT**			
miR-9	It's regulated by *c-myc* and targets *E-cadherin*	BRCA	Martello et al., 2010
miR-135b	It's regulated by hypoxia and regulates cell proliferation by modulating the hippo signaling pathway	CCC, HNSCC	Nagel et al., 2008; Lin et al., 2013
miR-210	Both miRNAs are being regulated by hypoxia and modulate TGF-β Signaling Pathway	BRCA, CRC	Huang et al., 2009; Volinia et al., 2012
miR-21			
miR-138	Modulates cell migration and invasion through targeting RhoC (Rho-related GTP-binding protein C) and ROCK2 (Rho-associated, coiled-coil-containing protein kinase 2)	HNSCC	Liu et al., 2011
MALAT1	Promotes activation of *LTBP3*, which at the same time regulates the bioavailability of *TGF-β*, a transduction signaling pathway for the transition. Also serves as sponge of miR-205.	Myeloma	Li B. et al., 2014
lncRNA H19	Modulates the expression of multiple genes involved in EMT by competing with miRNAs such as miR-138 and miR-200a, antagonizing their functions and stimulating the over-expression of *Vimentin, ZEB1, and ZEB2*.	CCC	Liang et al., 2015
**INFLAMMATION**			
miR-146b	Physiologically, is a target of *STAT3*, but in cancer its promoter is methylated, and consequently its down-modulation alters microRNA-mediated anti-inflammatory circuit.	BRCA	Xiang et al., 2014
lncRNA Lethe	Induced by pro-inflammatory cytokines via *NF-κB* or glucocorticoid receptor agonists, and functions in a negative feedback signaling with *NF-κB*.		Rapicavoli et al., 2013
lnc-IL7R	Diminishes the LPS-induced inflammatory response (E-selectin, *VCAM-1, IL-6*, and *IL-8*)		Cui H. et al., 2014

*CCC, Colorectal cancer; GBM, glioblastoma; HNSCC, Head and neck squamous cell carcinoma; PCA, Prostate Cancer; CML, Chronic myeloid leukemia; OvCa, Ovary cancer; BRCA, Breast cancer; GC, Gastric Cancer; ↑, up-expression; ↓, down-expression*.

Metabolism, mainly hypoxic conditions, can drive EMT through *NF*-κ*B, PI3K/Akt/mTOR, Notch, Wnt/*β-*catenin*, and Hedgehog signaling pathways (Fan et al., 2013). EMT refers to a complex molecular and cellular program by which epithelial cells lose their epithelial attributes such as cell–cell adhesion, planar-basal polarity, and limited motility, but acquire mesenchymal features, including increased motility, invasiveness and development of escape routes for apoptosis (Polyak and Weinberg, 2009). Modulation of EMT pathways by ncRNAs has been described in several tumors (Table 4). Another important feature that characterizes the most advanced and aggressive tumors is angiogenesis; meaning the development of tumor neovasculature. This mechanism is crucial to satisfy nutrient and oxygen demands, as well as to provide routes for metabolic waste excretion (Carmeliet and Jain, 2000). To achieve this oncogenic hallmark, tumor cells induce pro-angiogenic factors or block anti-angiogenic signals, in part by modulating ncRNA expression profiles (Table 4). For a more detailed overview about ncRNAs implicated in EMT and angiogenesis refer to Wang W. et al. (2015) and Choudhry et al. (2016).

Finally, inflammation is considered as an oncogenic feature that allows the acquisition of carcinogenic capacities by the provision of biomolecules to the tumor and to the cells of the microenvironment, such as transcription factors which can enhance proliferative signaling, pro-angiogenic factors, invasion and metastasis (Hanahan and Weinberg, 2011; Table 4). A more detailed overview of ncRNAs implicated in inflammation is discussed in (O'connell et al., 2012).

## Novel insights: ncRNAs as therapeutic tools in cancer metabolism

**Figure 5:**
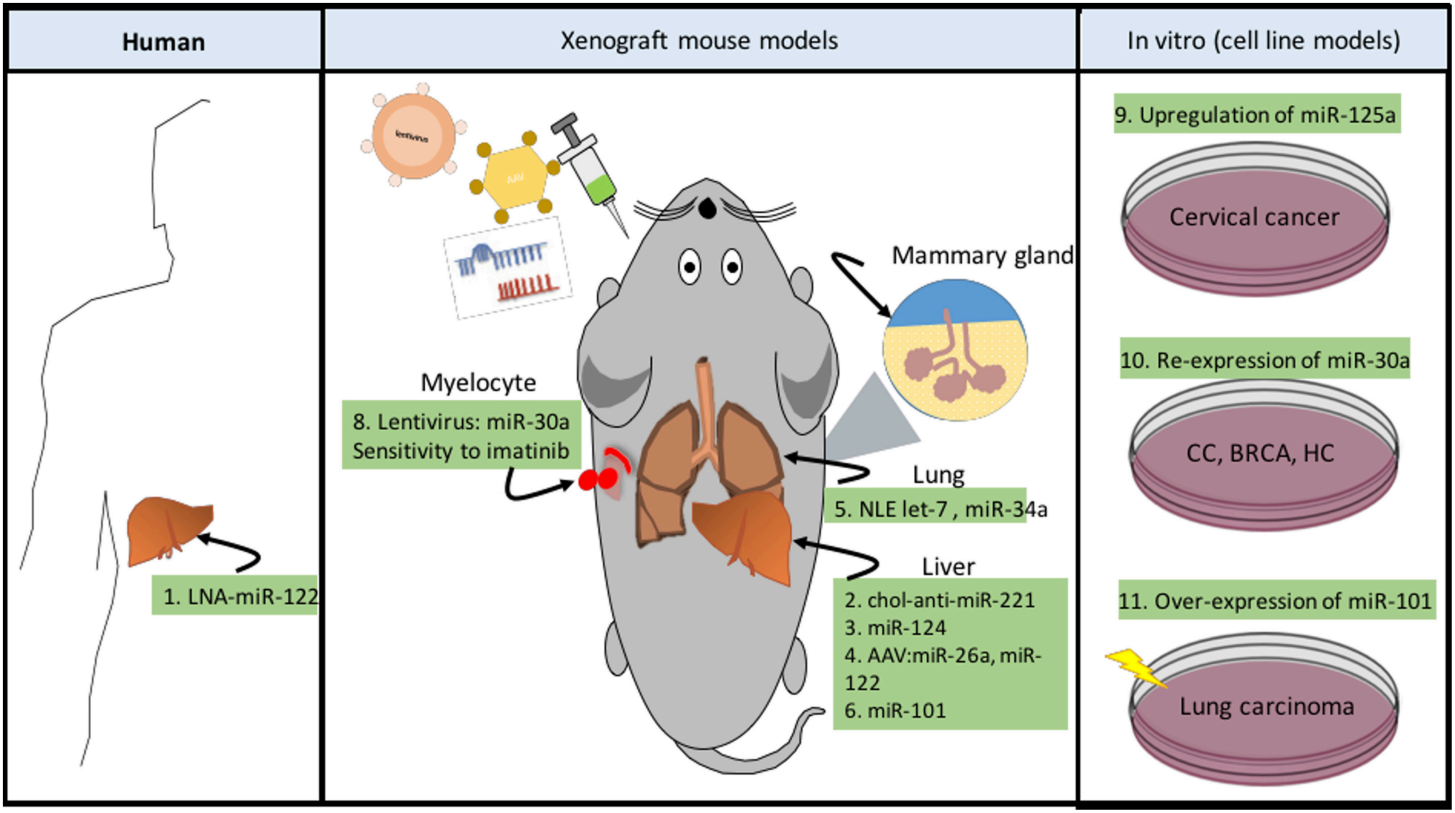
**ncRNAs as novel therapeutic strategies in cancer metabolism**. Targeting cancer metabolism represents a novel resource to develop anti-cancer therapies. Now a days, there are different techniques developed to specifically modulate metabolic pathways, some of them are dedicated to silencing (LNA) or re-expressing (miRNA mimic) ncRNA transcripts (Phan et al., 2014). These systems can be delivered by intratumoral, intraperitoneal, and intravenous injections, through systemic adenovirus-associated virus (AAV), or in complexes with neutral lipid emulsions (Drakaki et al., 2013). In addition to these technologies, cholesterol-modified miRNAs (chol-anti-miRs) exhibit improved pharmacokinetics and antitumor efficacy. *Human* (1) The development of hepatocellular carcinoma (HCC) in persons who are persistently infected with hepatitis C virus (HCV) is a growing problem. A phase II trial of the LNA anti-miR-122 is being carried out for treatment of HCV infection (Lindow and Kauppinen, 2012). *Xenograft mouse models* (2) chol-anti-miR-221 effectively suppresses liver tumor growth (Park et al., 2011). (3) Systemic administration of miR-124 suppresses liver cancer growth through suppression of the *IL6/STAT3* inflammatory pathway (Hatziapostolou et al., 2011). (4) AAV delivery of miR-26a or miR-122 suppresses *MYC*-driven liver carcinogenesis without affecting normal hepatocytes (Kota et al., 2009; Hsu et al., 2012). (5) Neutral lipid emulsions (NLE) to deliver let-7 which targets *RAS* and *MYC* oncogenes, as well as miR-34, reduces tumor size in lung cancer (Trang et al., 2011). (6) miR-101 and miR-376b are miRNAs, which negatively regulate the autophagy pathway (Frankel et al., 2011; Korkmaz et al., 2012). Furthermore, overexpression of miR-101 suppressed tumor development and efficiently reduced tumor size in liver cancer (Su et al., 2009). (7) Over-expression of miR-101 can effectively reduce tamoxifen-induced autophagy and enhance the sensitivity of breast cancer cells to tamoxifen treatment (Frankel et al., 2011). (8) Recombinant lentivirus administration of miR-30a (inhibitor of autophagy by down-modulating BECN1), can enhance sensitivity to imatinib cytotoxicity in chronic myeloid leukemia, increasing tumor cell apoptosis (Yu et al., 2012). *In vitro (cell line models)*. (9) Up-regulation of miR-125a in cervical cancer (CC) models sensitized to paclitaxel by down-regulating *STAT3* (Fan et al., 2016). (10) Re-expression of miR-30a can sensitize tumor cells to cisplatin via mediating autophagy in HeLa, MCF-7 and HepG2 (Zou et al., 2012). (11) Over-expression of miR-101 sensitized human lung carcinoma cells to radiation treatment (Yan et al., 2010).

The advent of novel knowledge and high throughput technologies, such as RNA-seq, Chip-seq, and metabolomic analysis, has allowed us to gain insight into the versatility of the mechanism that regulate metabolism and how the disturbance of specific factors, in particular ncRNAs, might impact the altered phenotypes of cancer cells. During the past years, we have gained important understanding about the biological activity of ncRNAs, although more research is needed to better understand the complex mechanisms that orchestrate tumor metabolism. Furthermore, pharmacological intervention of cell metabolism is emerging as a potential therapeutic strategy in some cancers (Ahn and Metallo, 2015) giving us the opportunity to explore new sources for biomarker discovery and development of new targeted drugs. The crucial role of ncRNAs in metabolism and associated mechanisms raises the possibility of developing ncRNA-targeted therapies. miRNA and lncRNAs mimics or inhibitors can be used to elevate or block the activity of metabolic-related genes to drive cancer initiation and/or progression programs. Figure 5 summarizes some of the actual and future therapeutic applications of metabolism-related ncRNAS in cancer treatment.

## Author contributions

SR and AH coordinated the review process, SR, AH, FB, and AC performed the literature review, organized the information and wrote the paper. All authors read and approved the last version of the manuscript.

### Conflict of interest statement

The authors declare that the research was conducted in the absence of any commercial or financial relationships that could be construed as a potential conflict of interest.
